# Ginkgolide C Alleviates Acute Lung Injury Caused by Paraquat Poisoning via Regulating the Nrf2 and NF-*κ*B Signaling Pathways

**DOI:** 10.1155/2022/7832983

**Published:** 2022-06-06

**Authors:** Rui Zhang, Cuirong Zhao, Xianwei Gong, Jie Yang, Guifang Zhang, Wen Zhang

**Affiliations:** Department of Pharmacy, Shandong Provincial Hospital Affiliated to Shandong First Medical University, Jinan, Shandong 250021, China

## Abstract

Paraquat (PQ), a highly toxic herbicide and primary attack for lung, results in severe acute lung injury (ALI) appeared as evident oxidative stress, inflammation, and apoptosis. Increasing evidence elucidates that nuclear factor erythroid-2-related factor 2 (Nrf2) and its associated nuclear factor-*κ*B (NF-*κ*B) exhibit many merits for protection of ALI by coordinating a fine-turned response to oxidative stress, inflammation, and apoptosis. Ginkgolide C (GC) has been reported to be a safe and potent therapeutic agent against ALI. However, whether GC could protect ALI induced by PQ poisoning and the possible underlining mechanisms have remained not to be fully elucidated. A rat model of ALI and a model of acute type II alveolar epithelial cell (RLE-6TN) injury constructed by exposure to PQ were applied to discuss the protective effect of GC. Furthermore, Nrf2 gene silencing RLE-6TN cells were used to discuss the exact mechanism. We confirmed that GC significantly ameliorated the histopathological damages, ultrastructural changes, lung injury score, W/D ratio, and Hyp activity of lung tissue and inhibited polymorphonuclear neutrophil (PMN) infiltration after PQ poisoning. Further results revealed that GC remarkably activated Nrf2-based cytoprotective system and inhibited NF-*κ*B-induced inflammatory injury as well as apoptosis. Taken together, we concluded that GC preserved protection of PQ-induced ALI via the Nrf2-NF-*κ*B dependent signal pathway, which may provide us novel insights into the treatment strategies for PQ poisoning.

## 1. Introduction

Paraquat (1,1′-dimethyl-4,4′-bipyridinium dichloride, PQ) is a wildly used herbicide with seriously high mortality because of its severe toxicity and lack of efficient rescue strategy [1–3]. In the past decades in China, the prevalence of PQ poisoning has remarkably increased because of its cheap and easy acquirement [4, 5]. Most of these patients died of acute lung injury (ALI), which attributed to selectively higher accumulation in lung tissue than that in other organs [6]. The specific toxic mechanism of PQ-induced ALI remains obscure, but it is generally accepted that PQ exhibits serious toxic effects via promotion of a redox cycling process that results in the eruption of acute inflammation. Therefore, clarifying the exact mechanism of PQ-induced ALI will help to discover an effective drug to relieve PQ poisoning symptoms and provide basis for studying other types of ALI or pulmonary fibrosis.

Recently, Nrf2, a member of Cap ‘n' Collar (CNC) subfamily, modulates a wide range of gene expression such as antioxidant proteins, stress response proteins, metabolic alteration enzymes, and detoxifying enzymes. Most of them apply critical process in the cellular defense system, particularly in modulation of oxidative stress [7–9]. Under normal conditions, Nrf2 predominately binds to the Kelch-like ECH-associating protein 1 (Keap1) in cytoplasm. Once activated by stress, Nrf2 dissociates from Keap1 and translocates into the nucleus, then heterodimerizes with small Maf proteins and binds to ARE, and finally activates a series of cytoprotective genes and regulates generation of target gene products, including various kinds of antioxidative enzymes [10]. At multiple levels, Nrf2 can exactly control the antioxidant defense by coordinately regulating these key components in the antioxidant system that, thus ensuring a timely and adequate response to oxidants.

Additionally, extensive researches have shown that Nrf2 was also critical in anti-inflammation except protecting against oxidative insults [11, 12]. Here, intensive researches have demonstrated that the mitigated inflammation induced by Nrf2 is related to the suppression of the nuclear factor-*κ*B (NF-*κ*B) pathway and downstream proinflammatory cytokines. In the latent state, NF-*κ*B is sequestered by its inhibitor of *κ*B (I*κ*B) protein in the cytosol [13]. Oxidative stress could trigger the phosphorylation of I*κ*B which results in the release and nuclear translocation of NF-*κ*B and activation of related cytokines and adhesion molecules [14]. Meanwhile, the accumulation and infiltration of polymorphonuclear neutrophils (PMNs) in both interstitial and alveolar spaces of the lungs, which in turn damages the respiratory function, is another important pathological feature of ALI [15].

With the persistent and extreme infiltration of PMNs, several toxic factors, such as reactive oxygen species (ROS), proinflammatory cytokines, and procoagulant molecules, may bring additional injury to lung, which finally aggravates ALI [16]. Further, the terminal result of seriously oxidative stress, inflammatory responses, and PMN infiltration is often the appearance of apoptosis [17]. Accordingly, targeting Nrf2 and NF-*κ*B signaling pathway has been considered a promising therapeutic strategy for prevention and reversal of PQ-induced ALI.

*Ginkgo biloba* has emerged as a traditional herbal remedy for thousands of years in China [18, 19]. An increasing body of evidence suggested that its leaf extracts had various biological properties such as cardioprotective effects, antineurovascular insults, and anticancer activities [20–22]. Ginkgolide C (GC) is a flavonoid monomer extracted from *Ginkgo biloba* leaf, and we previously discovered and identified that it could protect against ALI induced by LPS via inhibiting the CD40/NF-*κ*B signaling pathway [23]. However, it is still unclear whether GC could alleviate the ALI induced by PQ poisoning.

Therefore, the purpose of this study was to explore the protective effects of GC against PQ-induced ALI both *in vivo* and *in vitro* and to clarify the potential underlying mechanism. The present study may provide a novel therapeutic drug for PQ-induced ALI and further promote the application of GC in clinical treatment. Moreover, all the results might highlight the importance of Nrf2 in the modulation of the oxidative stress, inflammatory response, and apoptosis and demonstrate the possibility of complex crosstalk between Nrf2 and NF-*κ*B which will provide a feasible therapeutic target for the treatment of PQ-induced ALI.

## 2. Materials and Methods

### 2.1. Materials and Reagents

GC (PubChem CID: 161120) and PQ were obtained from Sigma-Aldrich (Sigma, MO, USA). Anti-Nrf2, anti-HO-1, anti-NQO-1, anti-GCLM, anti-ICAM-1, anti-VCAM-1, anti-iNOS, anti-IKK-*β*, anti-I*κ*B-*α*, anti-NF-*κ*B p65, anti-phosphorylated (p)-I*κ*B-*α*, anti-Bcl-2, anti-Bcl-xl, anti-Bax, anti-Caspase-3, anti-Caspase-9, anti-GAPDH, anti-*β*-actin, anti-histone, and IgG-HRP antibodies were products of Santa Cruz Biotechnology (Santa Cruz, Texas, USA). BCA protein concentration assay kit, PVDF membranes, and SDS-PAGE gel preparation kit were purchased from Beyotime Institute of Biotechnology. ECL plus kit was obtained from Nanjing KeyGen Biotech Co., Ltd. (KeyGen, Nanjing, CN). TNF-*α*, IL-1*β*, and IL-6 ELISA kits were obtained from Abcam (Cambrige, UK). GSH, NADPH, SOD, CAT, MDA, CK, and LDH kits were products of Nanjing Jiancheng Engineering Institute (Nanjing, CN).

### 2.2. Animals

40 adult male SD rats (weight, 200 ± 20 g; age, 8-10 weeks) were provided by Experimental Animal Center of Shandong First Medical University. All rats were kept in a controlled environment with temperature of 20-25°C, humidity 40-60%, and 12 h light-dark cycle and allowed to eat and drink freely. All the experiments were strictly implemented following the agreement of the Ethics Committee of the Shandong Provincial Hospital Affiliated to Shandong First Medical University.

### 2.3. In Vivo ALI Induction by PQ in Rats

40 rats were randomly divided into 5 groups (*n* = 8 per group) and treated as previously described [24, 25]: (1) control group, rats received no PQ but saline; (2) PQ group, rats were intragastric administration with 30 mg/kg PQ; (3) 8 mg/kg GC group, rats received 8 mg/kg GC after administration of 30 mg/kg PQ; (4) 16 mg/kg GC group, rats received 16 mg/kg GC after administration of 30 mg/kg PQ; and (5) 32 mg/kg GC group, rats received 32 mg/kg GC after administration of 30 mg/kg PQ. The GC groups received intraperitoneal injections of GC at the same time every day for 5 consecutive days.

### 2.4. Histopathological Assessment

The lung tissues were firstly fixed in 10% neutral-buffered formalin and embedded in paraffin via an automated processor. Then, a series of graded alcohols and xylene were used to process the lung tissues at 45°C for 10 min. 6 *μ*m thick tissue section was stained with hematoxylin and eosin (HE) at 37°C for 15 min. Histopathological observation was performed under a BX51 light microscope (Olympus Corporation; magnification, ×200). The number of PMNs was counted in three randomly fields.

### 2.5. Evaluation of Lung Injury Score

The lung injury score was applied to evaluate the severity of lung injury as previously described [23]. According to the thickness of alveolar wall, the amount of inflammatory infiltration, and the level of hemorrhaging, the lung score was calculated. Lung injury score was graded on a scale of 0 to 8 as follows: 0, no damage; 2, mild damage; 4, moderate damage; 6, severe damage; and 8, extremely severe damage.

### 2.6. Measurement of Lung Wet-to-Dry (W/D) Weight Ratio

The extent of lung edema/water accumulation was accessed by lung W/D weight ratio. After isolation of the lung from the rat, the wet lung weight was immediately measured. Then, the lung was dried at 60°C for 24 h to measure the dry lung weight.

### 2.7. Hydroxyproline (Hyp) Activity Assay

A Hyp Colorimetric Assay Kit (BioVision, Inc.) was applied to determine the Hyp activity in lung tissue according to the manufacturer's protocol.

### 2.8. Transmission Electron Microscopy

Before embedded in Spon 812 (SPI-Chem, Inc.; Structure Probe, Inc.) at 60°C for 48 h, the lung tissue was fixed with glutaraldehyde buffered fixative (3%; pH 7.2) at 25°C for 2-3 days. 60-80 nm lung tissue section was stained with 2% uranium acetate at 25°C for 10 min and subsequently lead citrate at 25°C for 5 min. Finally, the lung tissue section was washed with PBS and then analyzed under a JEM-2000EX transmission electron microscope (JEOL, Ltd.; magnification, ×17000) in three randomly fields.

### 2.9. Determination of Myeloperoxidase (MPO) Activity and PMN Infiltration Analysis

A MPO kit (Nanjing Jiancheng Bioengineering Institute) was applied to determine the MPO activity in lung tissues according to the manufacturer's protocol. MPO activity was expressed as U/g of protein.

### 2.10. Immunohistochemistry

Before embedded in paraffin at 60°C for 2 h, the lung tissue was fixed in 4% paraformaldehyde at 4°C for 2 h. Then, the lung tissue was blocked with 10% BSA (Sigma-Aldrich, Inc.) at 25°C for 30 min. Subsequently, 3-5 *μ*m lung tissue section was incubated with anti-ICAM-1, anti-VCAM-1, anti-iNOS, anti-NQO-1, and anti-GCLM antibodies (all 1 : 800) overnight at 4°C. The lung tissue section was then incubated with an anti-rabbit IgG secondary antibody (1 : 1000) at 25°C for 30 min and stained with 0.05% DAB at 25°C for 5 min. A fluorescence microscope (magnification, ×1000) was applied to perform the immunohistological analysis. The optical density (OD) of the positively stained area was quantified by Image-Pro Plus software (version 6; Media Cybernetics Inc.).

### 2.11. In Vitro Injury by PQ in Rat Type II Alveolar Epithelial Cells (RLE-6TN)

RLE-6TN cells were obtained from Shanghai Sixin Biotechnology Company (Shanghai, China). Then, the RLE-6TN cells were plated at a density of 4 × 10^4^ cells/mL in DMEM supplemented with 10% fetal calf serum. After 3 days, cells reaching 80% confluence were used for experiments.

RLE-6TN cells were randomly divided into 5 groups (*n* = 8 per group) and treated as previously described [24, 25]: (1) control group, cells were cultured in DMEM; (2) PQ group, cells were treated with 20 *μ*M PQ for 4 h; (3) 1 *μ*M GC group, cells were incubated with 1 *μ*M GC for 24 h after PQ treatment; (4) 10 *μ*M GC group, cells were incubated with 10 *μ*M GC for 24 h after PQ treatment; and (5) 100 *μ*M GC group, cells were incubated with 100 *μ*M GC for 24 h after PQ treatment.

### 2.12. Reconstruction of Nrf2 Gene Silencing RLE-6TN Cells

After the density of cells reached 1 × 10^5^/mL, cells in control group were transfected with pGPU6/Hygro and cells in other groups were transfected with pGPU6/Hygro-Nrf2 for 24 h using transfection reagent. The pGPU6/Hygro, pGPU6/Hygro-Nrf2, and transfection reagents were all products of Shanghai GenePharma Co., Ltd. (GenePharma, Shanghai, CN). After transfection, the Nrf2 gene silencing cells were cultured, modeled, and grouped according to the aforementioned method.

### 2.13. Cell Viability Assay

Cells in each group were incubated with 5 mg/ml MTT at 37°C for 4 h, and then, 100 *μ*L DMSO was added for 15 min to dissolve the formazan crystals. A microplate reader (Thermo Fisher Scientific, Inc.) was applied to determine the OD at the wavelength of 490 nm. The result was expressed as a percentage of the OD in control group.

### 2.14. ROS Assay

The level of ROS was determined by a 2′,7′-dichlorofluorescein diacetate (DCFDA, Abcam, USA) reagent. Cells were seeded into 6-well plates at a density of 1 × 10^5^ cells/well and incubated with 10 *μ*M DCFDA at 37°C for 20 min. The level of ROS was detected via a fluorescence microscope (BD Bioscience, USA). The fluorescence intensity of ROS was quantified by Image-Pro Plus software (version 6; Media Cybernetics Inc.).

### 2.15. Western Blotting

A nuclear and cytoplasmic protein extraction kit (Beyotime Institute of Biotechnology) was applied to extract the cytoplasmic and nuclear proteins as previous described [23]. And a BCA assay kit was applied to determine the protein concentration. 50 *μ*g proteins were separated by 10% SDS-PAGE and then transferred to a PVDF membrane (20 V; 100 mA) overnight. After blocking with 5% skimmed milk at 37°C for 4 h, the membrane was incubated with primary antibodies targeted against: Nrf2 (1 : 1000), HO-1 (1 : 1000), ICAM-1 (1 : 500), VCAM-1 (1 : 500), iNOS (1 : 500), NF-*κ*B p65 (1 : 1000), p-I*κ*B-*α*(1 : 1000), I*κ*B-*α* (1 : 500), IKK-*β* (1 : 500), Histone (1 : 1000), and *β*-actin (1 : 2000) at 4°C for 24 h. The membrane was then incubated with HRP-conjugated secondary antibodies (1 : 800) at 25°C for 12 h. ECL Plus kit and Gel Imaging System (Thermo Fisher Scientific, Inc.) were applied to visualize the protein bands. The expression level of protein was quantified by Quantity One software (version 4.0; Bio-Rad Laboratories, Inc.). *β*-Actin and Histone were used as the loading controls for cytoplasmic and nuclear proteins, respectively.

### 2.16. Measurement of GSH, NADPH, SOD, CAT, MDA, LDH, CK, TNF-*α*, IL-1*β*, and IL-6

Blood samples were obtained before the rats were sacrificed. And the supernatants of cells were collected from medium after PQ and GC treatment. The levels of GSH, NADPH, SOD, CAT, MDA, LDH, CK, TNF-*α*, IL-1*β*, and IL-6 were detected in blood samples and cell supernatants according to the manufacturer's instructions.

### 2.17. Statistical Analysis

Statistical analyses were performed by GraphPad Prism software (version 5.0; GraphPad Software, Inc.). Data were expressed as the mean ± SD of three independent experiments. Comparisons among groups were analyzed using one-way ANOVA followed by Bonferroni's post hoc test. *P* < 0.05 was considered to indicate a statistically significant difference.

## 3. Results

### 3.1. GC Improves Histopathological Injuries, Lung Injury Score, W/D Ratio, and Hyp Activity in PQ-Induced ALI Rat Model

**Figure 1:**
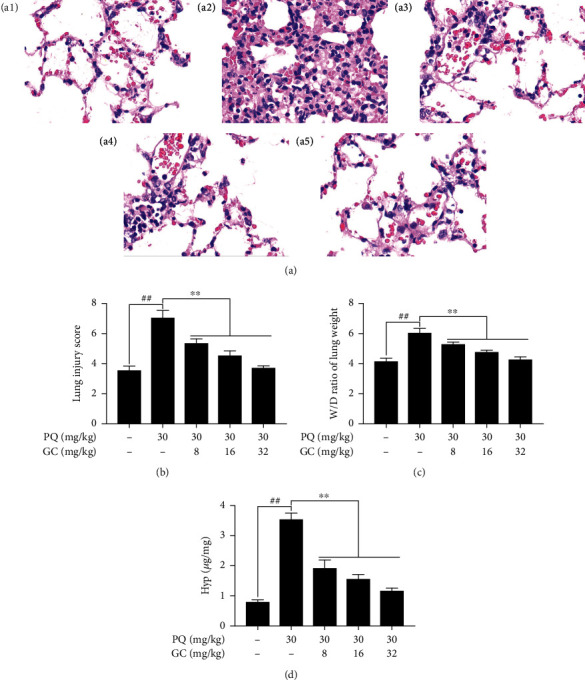
Effects of GC on histopathological alterations, lung injury score, W/D weight ratio, and Hyp activity in PQ-induced ALI rat model. (a) Histopathological morphology was evaluated by HE staining (magnification, ×200) in the control group (a1), PQ group (a2), 8 mg/kg GC group (a3), 16 mg/kg GC group (a4), and 32 mg/kg GC group (a5). Effects of GC on (b) lung injury score, (c) W/D weight ratio, and (d) Hyp activity. Data are presented as the mean ± S.D. (*n* = 8). ^##^*P* < 0.01 vs. the control group; ^∗^*P* < 0.05, ^∗∗^*P* < 0.01 vs. the PQ group.

As shown in Figure 1(a1), the lung tissue in the control group was normal, which represented as simple columnar epithelium and cuboidal epithelium respiratory bronchioles as well as normal wall structure in the pulmonary alveoli. On the contrary, there appeared severe pathological alterations in the PQ group, as indicated by significant cellular inflammatory infiltration, hemorrhage, pulmonary edema, and thickening of the alveolar walls with disorganized alveolar structure (Figure 1(a2)). Interestingly, treatment with 8, 16, or 32 mg/kg GC obviously improved PQ-induced histopathological injuries (Figures 1(a3)–1(a5)). Furthermore, PQ significantly increased the lung injury scores compared with the control group (Figure 1(b)). Nevertheless, 8, 16, or 32 mg/kg GC notably reversed PQ-induced high lung injury scores. Moreover, the lung W/D weight ratio was applied to access the degree of pulmonary edema in ALI induced by PQ. In the PQ group, the W/D weight ratio was obviously higher than that in the control group (Figure 1(c)). However, treatment with 8, 16, or 32 mg/kg GC could significantly alleviate the lung permeability as well as the alveolar epithelial barrier damage compared with the PQ group (Figure 1(c)). As shown in Figure 1(d), Hyp content of lung tissues in the PQ group was remarkably elevated compared with the control group (*P* < 0.01) and were lower in 8, 16, or 32 mg/kg GC groups than in the PQ group (*P* < 0.01).

### 3.2. GC Improves Pulmonary Ultrastructural Characterization and Inhibits the PMN Infiltration and MPO Activity in PQ-Induced ALI Rat Model

**Figure 2:**
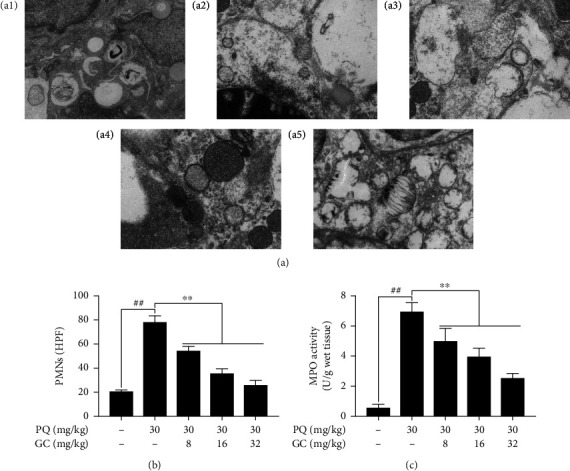
Effects of GC on ultrastructural characteristics, PMN count, and MPO activity in PQ-induced ALI rat model. (a) Transmission electron microscopy observation (magnification, ×17000) of lung injury in the control group (a1), PQ group (a2), 8 mg/kg GC group (a3), 16 mg/kg GC group (a4), and 32 mg/kg GC group (a5). Effects of GC on (b) PMN count and (c) MPO activity. Data are presented as the mean ± S.D. (*n* = 8). ^##^*P* < 0.01 vs. the control group; ^∗^*P* < 0.05, ^∗∗^*P* < 0.01 vs. the PQ group.

As shown in in Figure 2(a1), the endothelial cell did not show edema and vascular basement membrane was intact in the control group, whereas there were significant pathological alterations in the PQ group (Figure 2(a2)), including (i) extensive shedding of microvilli and empty lamellar bodies; (ii) vacuolization, degeneration, and necrosis in type I and II alveolar epithelial cells; and (iii) notable PMN infiltration, vascular endothelial cell edema, and basement membrane rupture in the alveolar spaces. Treatment with 8, 16, or 32 mg/kg GC remarkably alleviated PQ-induced endothelial cell damage. Additionally, PQ caused a significant increase of PMNs in the PQ group (*P* < 0.01 vs. the control group), which were all reversed by treatment with 8, 16, or 32 mg/kg GC (Figure 2(b)). And MPO activity was applied to evaluate the degree of neutrophilic infiltration. The results in Figure 2(c) revealed that 8, 16, and 32 mg/kg GC all significantly inhibited PQ-induced MPO activity.

### 3.3. GC Promotes Expressions of NQO-1 and GCLM and Prevents Upregulations of ICAM-1, VCAM-1, and iNOS in PQ-Induced ALI Rat Model

**Figure 3:**
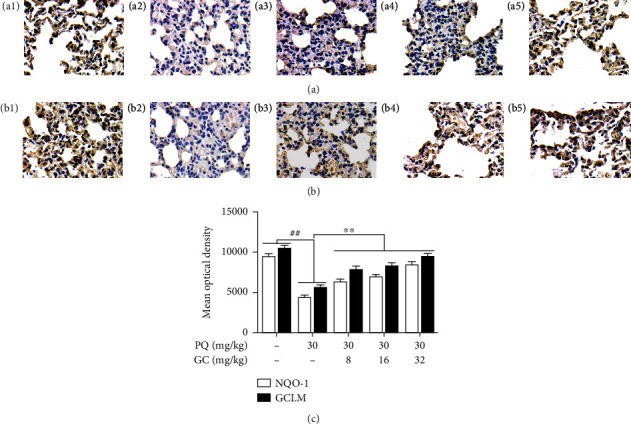
Effects of GC on NQO-1 and GCLM expressions in PQ-induced ALI rat model. Immunohistochemistry was applied to evaluate NQO-1 (a) and GCLM (b) expressions in lung tissue of the control group (a1/b1), PQ group (a2/b2), 8 mg/kg GC group (a3/b3), 16 mg/kg GC group (a4/b4), and 32 mg/kg GC group (a5/b5) (magnification, ×1000). The mean optical density of protein in each group (c). Data are presented as the mean ± S.D. (*n* = 8). ^##^*P* < 0.01 vs. the control group; ^∗∗^*P* < 0.01 vs. the PQ group.

In the control group, the levels of NQO-1 and GCLM were relatively high and the expressions of ICAM-1, VCAM-1, and iNOS were relatively low (Figures 3(a1), 3(b1), 4(a1), 4(b1), and 4(c1)). As shown in Figures 3(a2), 3(b2), 4(a2), 4(b2), and 4(c2), the expressions of NQO-1 and GCLM were significantly decreased but ICAM-1, VCAM-1, and iNOS expression levels were obviously elevated in the PQ group (*P* < 0.01). But 8, 16, and 32 mg/kg GC obviously upregulated NQO-1 and GCLM expression levels and downregulated ICAM-1, VCAM-1, and iNOS expression levels, compared with the PQ group (*P* < 0.01).

### 3.4. GC Increases the Expression Levels of HO-1, Elevates the Nuclear Translocation of Nrf2, and Represses the Nuclear Translocation of NF-*κ*B in PQ-Induced ALI Rat Model

**Figure 5:**
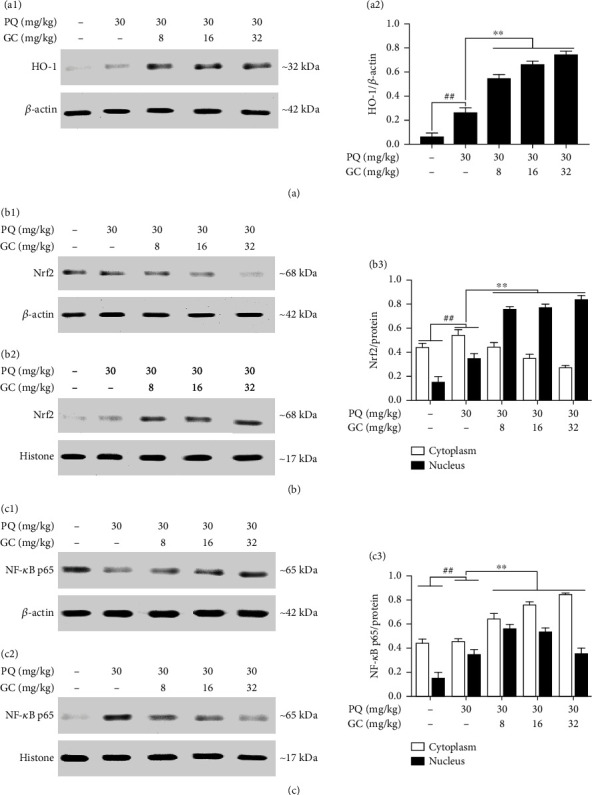
Effects of GC on HO-1 expression and nuclear translocations of Nrf2 and NF-*κ*B p65 in PQ-induced ALI rat model. (a1) GC obviously elevated the expression of HO-1. (b) GC obviously elevated the translocation of Nrf2 from cytosolic (b1) to nuclear (b2). (c) GC restrained the translocation of NF-*κ*B p65 from cytosolic (c1) to nuclear (c2). The levels of Nrf2 and NF-*κ*B p65 were separately determined in cytosolic and nuclear extracts. Results were expressed as Protein/reference protein ratio in each group (a2/b3/c3). Data were expressed as mean ± S.D. of three independent experiments. ^##^*P* < 0.01 vs. the control group; ^∗^*P* < 0.05, ^∗∗^*P* < 0.01 vs. the PQ group.

As shown in Figures 5(a1) and 5(a2), HO-1 expression in the control group was low and PQ slightly increased the level of HO-1 (*P* < 0.01 vs. the control group). Administration with 8, 16, or 32 mg/kg GC significantly increased the levels of HO-1 by 1.08-fold, 1.51-fold, and 1.83-fold, respectively, compared with the PQ group (all *P* < 0.01). In the control group, the levels of Nrf2 and NF-*κ*B p65 were relatively low the in nucleus but high in the cytoplasm (Figures 5(b1), 5(b2), 5(c1), and 5(c2)), whereas the nuclear translocation of Nrf2 from cytosol to the nucleus was slight but NF-*κ*B p65 was obvious in the PQ group compared with the control group (*P* < 0.01). Interestingly, 8, 16, or 32 mg/kg GC significantly promoted the translocation of Nrf2 but repressed the translocation of NF-*κ*B p65 after PQ treatment (Figures 5(b1)–5(b3) and 5(c1)–5(c3)).

### 3.5. GC Reduces Oxidative Stress and Inflammatory Damages via Enhancement of Antioxidant Capacity and Inhibition of Inflammatory Reaction in PQ-Induced ALI Rat Model

The results in Table 1 showed that the levels of GSH, SOD, and CAT were high and the levels of NADPH, MDA, LDH, CK, TNF-*α*, IL-1*β*, and IL-6 were low in the control group. But in the PQ group, the levels of GSH, SOD, and CAT were remarkably decreased by 71.47%, 51.75%, and 58.46% and the levels of NADPH, MDA, LDH, CK, TNF-*α*, IL-1*β*, and IL-6 were significantly increased by 0.88-fold, 3.47-fold, 1.74-fold, 2.49-fold, 10.86-fold, 7.96-fold, and 4.70-fold, respectively, compared with the control group (all *P* < 0.01). However, 8, 16, or 32 mg/kg GC significantly elevated the levels of GSH, SOD, and CAT and depressed the levels of NADPH, MDA, LDH, CK, TNF-*α*, IL-1*β*, and IL-6 in a dose dependent manner.

### 3.6. GC Enhanced Cell Viability and Decreased ROS Production in PQ-Injured RLE-6TN Cells

**Figure 6:**
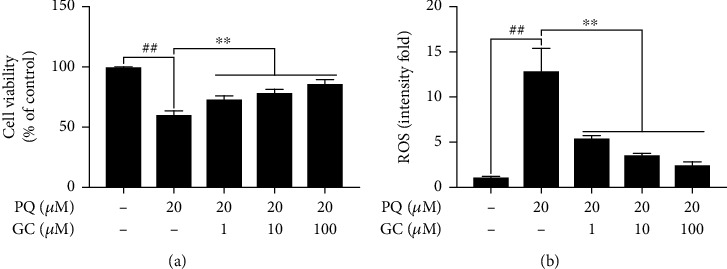
Effects of GC on cell viability and ROS production in PQ-injured RLE-6TN cells. GC significantly increased cell viability (a) and decreased ROS production (b). Data were expressed as mean ± S.D. of three independent experiments. ^##^*P* < 0.01 vs. the control group; ^∗∗^*P* < 0.01 vs. the PQ group.

As shown in Figure 6(a), the cell viability in the PQ group was significantly reduced to 60.14 ± 3.33% (*P* < 0.01 vs. the control group). 1, 10, or 100 *μ*M GC significantly improved cell viability in PQ-treated RLE-6TN cells (72.92 ± 3.10, 78.55 ± 2.88, and 85.93 ± 3.52%, respectively; all *P* < 0.01). Then, DCFH-DA was applied to determine the activity of ROS in RLE-6TN cells (Figure 6(b)). The level of ROS was obviously low in the control group but exposed to PQ showed a remarkable increase. However, 1, 10, and 100 *μ*M GC remarkably decreased the levels of ROS compared to the PQ group (all *P* < 0.01).

### 3.7. GC Enhances Antioxidant Capacity, Inhibits Inflammatory Reaction, and Resists Apoptosis in PQ-Injured RLE-6TN Cells

**Figure 7:**
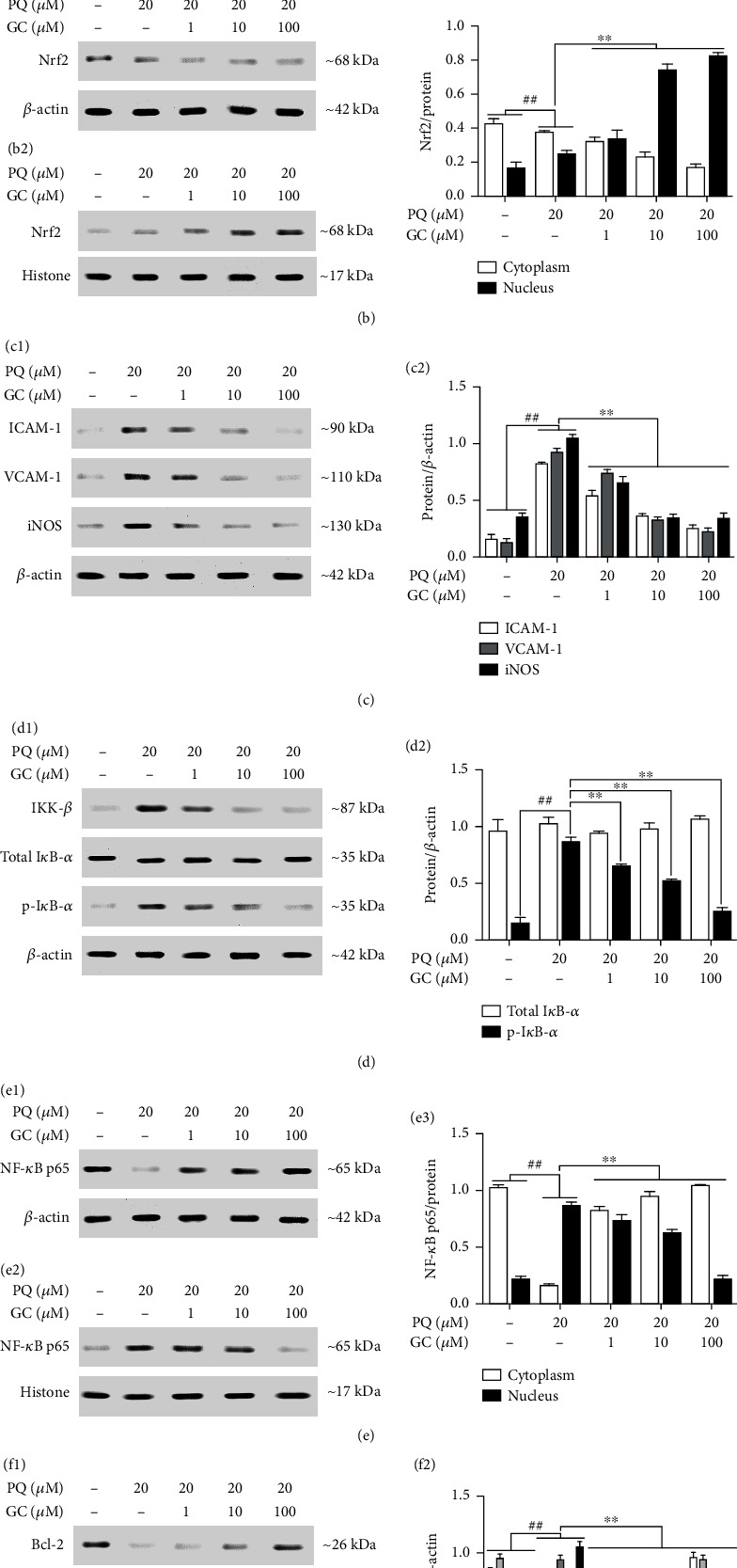
Effects of GC on expressions of HO-1, NQO-1, GCLM, ICAM-1, VCAM-1, iNOS, IKK-*β*, p-I*κ*B-*α*, Bcl-2, Bcl-xl, Bax, caspase-3, caspase-9, and nuclear translocations of Nrf2 and NF-*κ*B p65 by Western blot in PQ-injured RLE-6TN cells. (a1) GC increased the expressions of HO-1, NQO-1, and GCLM after PQ treatment. (b) GC increased the translocation of Nrf2 from cytosolic (b1) to nuclear (b2). (c1) GC reduced the expressions of ICAM-1, VCAM-1, and iNOS. (d1) GC inhibited the expressions of IKK-*β* and p-I*κ*B-*α*. (e) GC restrained the translocation of NF-*κ*B p65 from cytosolic (e1) to nuclear (e2). (f1) GC increased the expressions of Bcl-2 and Bcl-xl and reduced the expression of Bax, caspase-3, and caspase-9. The levels of Nrf2 and NF-*κ*B p65 were separately determined in cytosolic and nuclear extracts. Results were expressed as protein/reference protein ratio in each group (a2/b3/c2/d2/e3/f2). Data were expressed as mean ± S.D. of three independent experiments. ^##^*P* < 0.01 vs. the control group; ^∗∗^*P* < 0.01 vs. the PQ group.

ALI induced by PQ is a composite of multifarious events which starts with oxidative stress, inflammatory response, intracellular Ca^2+^ overload, and subsequent apoptosis with irreversible cell death. And, increased nuclear of Nrf2 would provoke the expressions of cytoprotective target genes such as HO-1, NQO1, and GCLM. Figures 7(a1) and 7(a2) showed that the expressions of HO-1, NQO1, and GCLM in the PQ group were slightly increased compared with the control group (all *P* < 0.01). Moreover, 1, 10, and 100 *μ*M GC could result in further significant increases of HO-1, NQO1, and GCLM compared with the PQ group (all *P* < 0.01). Moreover, 1, 10, and 100 *μ*M GC also clearly upregulated Nrf2 translocation from the cytosol into nucleus (Figures 7(b1)–7(b3)). Then, we explored the effect of GC on the key antioxidant enzymes. As illustrated in Table 2, GSH, SOD, and CAT activities in the control group were very high and NADPH, MDA, LDH, and CK activities were very low. Conversely, PQ treatment resulted in significant drop of GSH, SOD, and CAT activities and growth of NADPH, MDA, LDH, and CK activities, while treatment of 1, 10, and 100 *μ*M GC dose dependently reversed this trend.

During the period of ALI, new pathophysiological changes occurred, characterized by release of inflammatory factors and subsequent initiation of inflammatory cascade. Thus, we further measure the levels of ICAM-1, VCAM-1, and iNOS (Figures 7(b1) and 7(b2)) and the levels of TNF-*α*, IL-1*β*, and IL-6 (Table 2). PQ treatment triggered the eruption of inflammatory responses, whereas treatment of 1, 10, and 100 *μ*M GC validly reduced the amount of these inflammatory factors. On the basis that these inflammatory factors include strong connections with NF-*κ*B which can be inhibited by Nrf2, the effect of GC on the NF-*κ*B signal pathway was also discussed. The results in Figures 7(d1) and 7(d2) showed that PQ treatment induced notably elevated the activity of IKK-*β* and the phosphorylation of I*κ*B compared to the control group, which suggested the NF-*κ*B was stimulated in a canonical way. But the amount of total I*κ*B-*α* in each group was the same. Then, we investigated the nuclear translocation of NF-*κ*B and discovered that levels of NF-*κ*B p65 in nuclear were notably increased but these in cytoplasm were greatly reduced in the PQ group (Figures 7(e1)–7(e3)). However, these effects were effectively reversed by treatment with 1, 10, and 100 *μ*M GC.

With the continuous interweaving and sustained of oxidative stress and inflammatory response induced by PQ poisoning, subsequently, cell apoptosis appeared. As shown in Figures 7(f1) and 7(f2), the expressions of Bcl-2 and Bcl-xl were markedly decreased, but the expressions of Bax, caspase-3, and caspase-9 were remarkably increased in the PQ group (all *P* < 0.01 vs. the control group). Inversely, treatment with 1, 10, and 100 *μ*M GC eliminated the decrease of Bcl-2, Bcl-xl, and the rise of Bax, caspase-3, and caspase-9, respectively (all *P* < 0.01 vs. the PQ group).

### 3.8. GC Had No Effect on Cell Viability and ROS Production and Could Not Promote Antioxidant Capacity, Restrain Inflammatory Reaction, or Inhibit Apoptosis in PQ-Injured RLE-6TN Cells after Nrf2 Gene Silencing

**Figure 8:**
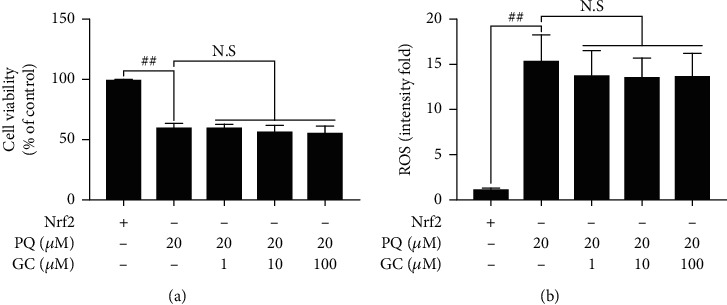
Effects of GC on cell viability and ROS production in PQ-injured Nrf2 gene silencing RLE-6TN cells. GC had no effect on the cell viability (a) and the levels of ROS (b) after PQ treatment in Nrf2 gene silencing RLE-6TN cells. Data were expressed as mean ± S.D. of three independent experiments. ^##^*P* < 0.01 vs. the control group; ^∗∗^*P* < 0.01 vs. the PQ group.

**Figure 9:**
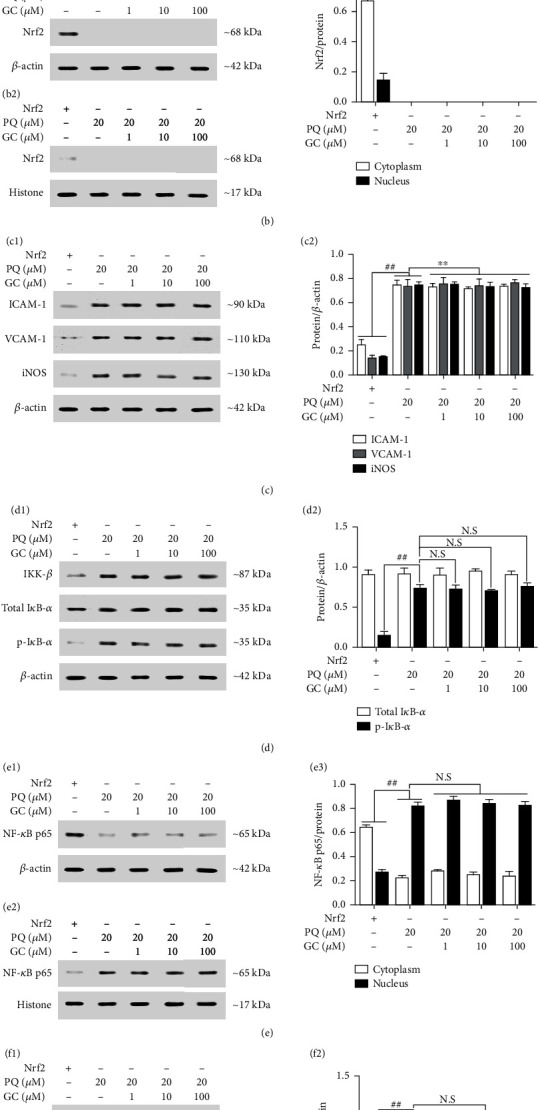
Effects of GC on expressions of HO-1, NQO-1, GCLM, ICAM-1, VCAM-1, iNOS, IKK-*β*, p-I*κ*B-*α*, Bcl-2, Bcl-xl, Bax, caspase-3, and caspase-9 and nuclear translocations of Nrf2 and NF-*κ*B p65 by Western blot in PQ-injured Nrf2 gene silencing RLE-6TN cells. GC had no effect on expressions of HO-1, NQO-1, and GCLM (a1); nuclear translocation of Nrf2 (b1/b2); expressions of ICAM-1, VCAM-1, and iNOS (c1); expressions of IKK-*β* and p-I*κ*B-*α* (d1); nuclear translocation of NF-*κ*B p65 (e1/e2); and expressions of Bcl-2, Bcl-xl, Bax, caspase-3, and caspase-9 (f1). The levels of Nrf2 and NF-*κ*B p65 were separately determined in cytosolic and nuclear extracts. Results were expressed as protein/reference protein ratio in each group (a2/b3/c2/d2/e3/f2). Data were expressed as mean ± S.D. of three independent experiments. ^##^*P* < 0.01 vs. the control group.

In order to further verify the key role of Nrf2 in the protective effect of GC on PQ-induced ALI. Reconstruction of Nrf2 gene silencing was applied to establish *in vitro* PQ-injured RLE-6TN cell model. The results in Figures 8(a) and 8(b) demonstrated that 12, 24, and 48 mg/kg GC all failed to increase the cell viability and decrease the ROS level against PQ injury after Nrf2 gene was silenced. More interestingly, consistent with the results of the *in vivo* studies, it was no accident that GC had no impact on the expressions of oxidative, inflammatory, and apoptotic factors, as well as NF-*κ*B translocation after Nrf2 gene silencing (Figures 9(a)–9(f) and Table 3).

## 4. Discussion

One major consequence of PQ poisoning is ALI, which normally results in high morbidity and frequently in death. The eruption of oxidative stress and the augmentation of inflammatory responses into the lung tissues, which could stimulate the production of numerous cytotoxic substances, such as various ROS, proinflammatory cytokines, and granular enzymes, and eventually irreversible lung injury or cell apoptosis [26], while interactions between Nrf2 and NF-*κ*B remarkably influence the modulation of oxidative stress and inflammatory response have been widely recognized [27]. Therefore, regulating the Nrf2/NF-*κ*B signaling pathway may provide a promising and effective therapy for PQ-induced ALI. As far as we know, this is the first study demonstrating the protective effect of GC on PQ-induced ALI and the underlying mechanism both *in vivo* and *in vitro*.

GC has been known to be involved in a wide range of biological and pharmacological functions against a variety of diseases such as ischemic heart disease, arrhythmias, cancer, diabetes, and cognitive disorders. Our previous researches have only studied the protection of GC to LPS-induced ALI but whether GC plays an effective role in PQ poisoning induced ALI is not clear. In present study, we first confirmed that treatment of GC at 8, 16, or 32 mg/kg could markedly alleviate PQ-induced ALI, characterized by significant improvements in histopathological injuries, reductions in lung injury scores, W/D ratio, and Hyp activity and repaired lung ultrastructure and alleviation in PMN infiltration as well as MPO activity. These significant changes can partially be explained by the fact that, GC could elicit the greatest protective effect on PQ-induced ALI.

Importantly, more recent researches have expounded that Nrf2 played an essential role in the regulation of oxidative stress and Nrf2 activation is a key event throughout the development of PQ poisoning [28]. Based on the in *vivo* and *in vitro* results, we also proved that Nrf2 was mainly in the cytoplasm of cells rather than in nucleus under unstressed conditions. But, when eruption of oxidative or xenobiotic stresses induced by PQ occurred, Nrf2 would rapidly accumulate and then translocate into the nucleus to form a heterodimer with the small Maf proteins. Most interestingly, our study also found that GC could significantly increase the nuclear translocation of Nrf2 in a concentration-dependent manner after PQ poisoning. Subsequently, Nrf2 binds to the regulatory regions of target genes and coordinately manages a variety of ARE/EpRE-driven genes which have important roles in regulating endogenous resistance to various stressors [29]. The most critical antioxidant gene activated by Nrf2 is HO-1 which can yield equimolar amounts of free divalent iron, biliverdin-Ix*α*, and carbon monoxide and catalyze the oxidative cleavage of the *α*-mesocarbon of Fe-protoporphyrin-IX [30]. Another essential antioxidant gene activated by Nrf2 is the NQO-1 gene which can produce an enzyme to prevent semiquinone redox cycling and the consequent oxidative stress [31]. In addition, GCLM has often been described as a modulatory subunit of glutamate-cysteine ligase [32]. Therefore, to determine the effects of GC on the expressions of the Nrf2-driven genes both *in vivo* and *in vitro*, HO-1, NQO-1, and GCLM, were detected by immunohistochemistry staining and Western blot. As expected, treatment of GC could induce a remarkable dose-dependent increases of HO-1, NQO-1, and GCLM both in lung tissues and in RLE-6TN cells, which further confirmed that GC could specifically activate Nrf2. Moreover, we investigated whether GC could improve the antioxidant capacity against PQ-induced oxidative stress both *in vivo* and *in vitro*. It is widely accepted that persistent redox cycling of PQ results in the continued depletion of NADPH and decrease of GSH activity, which eventually led to eruption of ROS generation. Therefore, GSH, NADPH, SOD, CAT, MDA, LDH, and CK were selected to discuss the impact of GC on the key oxidant enzymes. The enzyme activities of GSH, SOD, and CAT were significantly enhanced but NADPH, MDA, LDH, and CK were remarkably decreased by treatment of GC in a concentration-dependent manner. Collectively, these results confirmed that GC could obviously improve the antioxidant capacity of RLE-6TN cells injured by PQ poisoning.

A wealth of researches strongly suggested that imbalanced overproduction of ROS induced by oxidative stress promoted severe damage to lung tissue during the whole pathological processes of ALI [33]. We also found that an evident increase of ROS occurred in PQ-injured RLE-6TN cells, whereas GC treatment markedly reduced the level of ROS. This finding indicated that GC could exert antioxidative stress effect via rebalancing the production of ROS.

Furthermore, increasing evidence has showed that the remarkable augmentation of ROS as well as inflammatory factors were tightly intertwined and implicated in the pathogenesis of ALI [34]. Consistently, there is growing evidence of a crosstalk between the Nrf2 and inflammation pathways at different levels. Notably, NF-*κ*B is an essential transcription factor, which could be activated by overproduction of ROS and effectively regulate inflammation and cell apoptosis [35, 36]. In addition, recently published data has shown that upregulation of Nrf2 could enhance the inhibition of NF-*κ*B pathway as well as its dependent inflammatory responses [37]. And, this evidence has been further supported by this study that PQ poisoning could strongly elevate such nuclear translocations of NF-*κ*B, whereas GC could effectively inhibit the activation of NF-*κ*B pathway that has already been clearly proved in our previous study. Likewise, under normal conditions, I*κ*B family bind to NF-*κ*B proteins as components of inactive cytoplasmic complexes. However, numerous studies have proposed that Nrf2 could seriously disturb the phosphorylation of I*κ*B [38]. Therefore, we then conducted an association analysis of GC on phosphorylation of I*κ*B-*α*. Indeed, treatment with GC in RLE-6TN cells could dramatically suppress the notable phosphorylation of I*κ*B-*α* induced by PQ poisoning, which might be related to excessive activation of Nrf2 by GC. Perhaps, this significant change could partially be explained by the fact that excessive activation of Nrf2 by GC would lead to inhibition of I*κ*B-*α* phosphorylation, further constrain of NF-*κ*B translocation. In addition, several proinflammatory factors and adhesion molecules are excessively produced after NF-*κ*B activation triggered by oxidative stress. In the present study, we found that the expressions of TNF-*α*, IL-1*β*, IL-6, ICAM-1, VCAM-1, and iNOS were remarkably increased after PQ poisoning. Besides that, oxidative stress in turn initiates further activation of NF-*κ*B and overproduction of inflammatory factors. Along with these lines, we showed that GC treatment prevented the remarkable growth of these inflammatory cytokines both *in vivo* and *in vitro*, demonstrating downregulation of inflammatory responses involved in the activation of Nrf2.

Accumulating evidence has demonstrated that apoptosis is also one of the most important injury features of ALI [39]. Excessive generation of ROS and accumulation of inflammatory factors could cause serious mitochondrial dysfunction, followed by initiation of apoptosis triggered by activation of executioners of apoptosis such as caspase-3 and caspase-9 [40]. Our results confirmed that PQ poisoning indeed significantly increased the expressions of caspase-3 and caspase-9 in RLE-6TN cells. Nevertheless, treatment with GC effectively reverse this trend. In addition, Bcl-2, Bcl-xl, and Bax proteins are also involved in mitochondrial-dependent apoptosis. Interestingly, our present study confirmed that GC treatment also significantly promoted the expressions of Bcl-2 and Bcl-xl and prevented the increases of Bax in PQ-treated RLE-6TN cells. Altogether, these results suggested that GC could alleviate PQ-induced apoptosis that may be related to modulation of the Nrf2-dependent NF-*κ*B pathway.

Importantly, the major objective of this study was to explore whether anti-PQ-induced ALI property of GC was Nrf2-dependent; then, we silenced Nrf2 gene *in vitro* to validate our hypothesis. After Nrf2 was silenced, GC failed to improve the cell viability, ROS, antioxidant enzymes, NF-*κ*B pathway-induced inflammatory responses, and cell apoptosis. These findings provided support for a larger body of knowledge, which linked Nrf2 to protective effect of GC against PQ-induced ALI. All of these results highlighted the importance of Nrf2 in the modulation of the oxidative stress, inflammatory response, and apoptosis during PQ poisoning and demonstrated the possibility of complex crosstalk between Nrf2 and NF-*κ*B. However, PQ poisoning will directly cause damages to a variety of cells such as endothelial cells, vascular smooth muscle cells, mesenchymal stem cells, and other immune cells. In this study, we just focused on the effect of GC in PQ-injured alveolar epithelial cells. Whether GC has protective effect on other cells needs further discussion. Moreover, our previous research reported that GC, a specific CD40 inhibitor, could exhibit significant protective effect of myocardial ischemia and reperfusion injury via suppressing the CD40/NF-*κ*B signal pathway. Whether there is an association between CD40 and Nrf2 is unclear, and the effect of GC on them needs to be further studied. Therefore, we need to conduct further research to clarify the above problems.

PQ poisoning has become one of the most common pesticide poisoning in China. There are still no specific antidotes for PQ poisoning at home and abroad, and the death rate of PQ oral poisoning is up to 95%. Only routine treatments such as gastric lavage, intentional diarrhea, diuresis, and blood perfusion were adopted in clinical rescue of PQ poisoning. However, the therapeutic effect is disappointing, and the case fatality rate keeps high. It has become one of the hot issues for emergency medicine study to search PQ's special efficiency measures. Therefore, GC, which could significantly alleviate PQ-induced ALI, has good clinical research value and application prospect. This study will provide a new exploration direction and treatment idea for basic research and clinical treatment of PQ.

## 5. Conclusion

**Figure 10:**
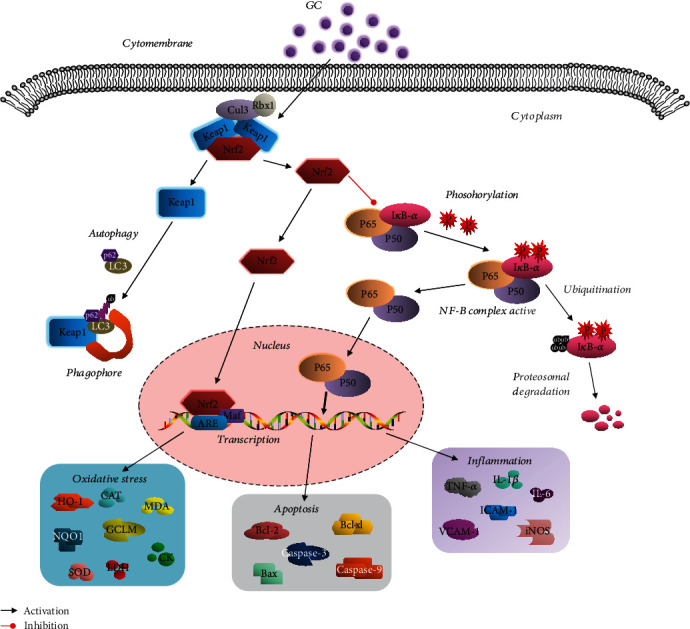
Schematic diagram describing the mechanism. GC could activate Nrf2-regulated antioxidative pathways and inhibit NF-*κ*B-dependent inflammatory response and apoptosis to exert the protective effect against PQ-induced ALI. 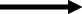 activation; 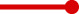 inhibition.

As described in Figure 10, GC, a potent Nrf2 activator, which dramatically activates Nrf2-regulated antioxidative pathways and inhibits NF-*κ*B-dependent inflammatory response and apoptosis to exert the protective effect against PQ-induced ALI both *in vivo* and *in vitro*, unveils therapeutic opportunities against PQ poisoning.

## Figures and Tables

**Figure 4:**
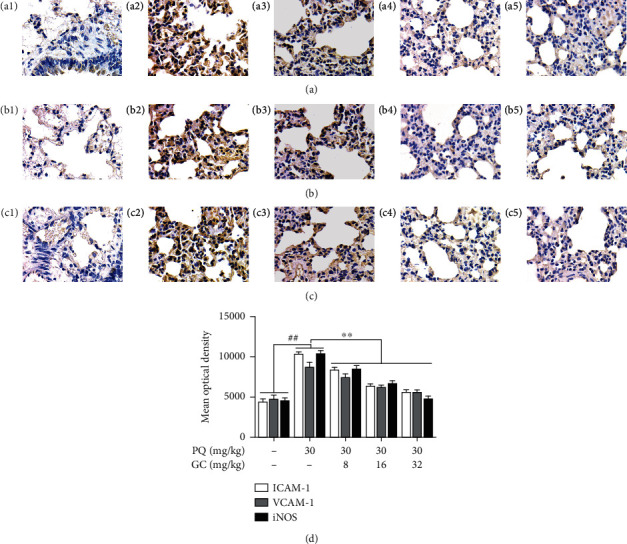
Effects of GC on ICAM-1, VCAM-1, and iNOS expressions in PQ-induced ALI rat model. Immunohistochemistry was applied to evaluate ICAM-1 (a), VCAM-1 (b), and iNOS (c) expressions in the lung tissue of the control group (a1/b1/c1), PQ group (a2/b2/c2), 8 mg/kg GC group (a3/b3/c3), 16 mg/kg GC group (a4/b4/c4), and 32 mg/kg GC group (a5/b5/c5) (Magnification, ×1000). The mean optical density of protein in each group (d). Data are presented as the mean ± S.D. (*n* = 8). ^##^*P* < 0.01 vs. the control group; ^∗∗^*P* < 0.01 vs. the PQ group.

**Table 1:** Effects of GC on antioxidant enzymes and inflammatory cytokines in PQ-induced ALI rat model.

Group	Control	PQ	PQ + GC		
Dose (mg/kg)		30	8	16	32
GSH (*μ*M)	258.15 ± 27.27	73.65 ± 14.07^**##**^	137.91 ± 28.80^∗∗^	168.48 ± 14.97^∗∗^	188.66 ± 21.30^∗∗^
NADPH (*μ*M)	3.49 ± 0.37	6.57 ± 0.26^**##**^	5.61 ± 0.23^∗∗^	4.58 ± 0.30^∗∗^	3.95 ± 0.31^∗∗^
SOD (U/mL)	143.60 ± 22.34	69.29 ± 6.23^**##**^	95.83 ± 2.86^∗∗^	118.98 ± 2.79^∗∗^	140.04 ± 5.76^∗∗^
CAT (U/mL)	8.83 ± 0.58	3.67 ± 0.77^**##**^	5.29 ± 0.52^∗∗^	6.50 ± 0.29^∗∗^	7.29 ± 0.31^∗∗^
MDA (nM)	1.71 ± 0.14	7.63 ± 0.78^**##**^	5.32 ± 0.62^∗∗^	4.02 ± 0.46^∗∗^	2.52 ± 0.30^∗∗^
LDH (U/L)	3690.37 ± 117.49	10105.00 ± 792.10^**##**^	7064.54 ± 699.47^∗∗^	6188.48 ± 506.88^∗^	3835.30 ± 544.71^∗∗^
CK (U/mL)	0.52 ± 0.11	1.81 ± 0.16^**##**^	1.26 ± 0.12^∗∗^	0.80 ± 0.12^∗∗^	0.66 ± 0.08^∗∗^
TNF-*α* (pg/mL)	8.26 ± 0.81	97.95 ± 6.83^**##**^	61.17 ± 7.05^∗∗^	39.63 ± 6.69^∗∗^	23.43 ± 4.64^∗∗^
IL-1*β* (pg/mL)	31.32 ± 5.63	280.55 ± 9.12^**##**^	183.43 ± 13.40^∗∗^	79.16 ± 15.04^∗∗^	43.31 ± 10.59^∗∗^
IL-6 (pg/mL)	15.79 ± 2.92	89.95 ± 5.32^**##**^	57.95 ± 3.64^∗∗^	39.38 ± 6.18^∗∗^	26.07 ± 2.49^∗∗^

Values were expressed as mean ± S.D. (*n* = 8). ^##^*P* < 0.01 vs. the control group; ^∗^*P* < 0.05, ^∗∗^*P* < 0.01 vs. the PQ group.

**Table 2:** Effects of GC on antioxidant enzymes and inflammatory cytokines in PQ-induced RLE-6TN model.

Group	Control	PQ	PQ+GC		
Dose (*μ*M)		20	1	10	100
GSH (mg/g pro)	2.82 ± 0.43	1.56 ± 0.37^**##**^	1.73 ± 0.16^∗^	2.27 ± 0.16^∗∗^	2.63 ± 0.09^∗∗^
NADPH (*μ*M)	13.90 ± 3.62	27.53 ± 1.89^**##**^	22.57 ± 1.67^∗∗^	17.33 ± 0.94^∗∗^	14.95 ± 3.08^∗∗^
SOD (U/mg pro)	336.81 ± 32.20	149.11 ± 36.49^**##**^	205.49 ± 9.34^∗∗^	227.81 ± 18.06^∗∗^	267.55 ± 10.25^∗∗^
CAT (U/mL)	6.05 ± 0.43	1.55 ± 0.22^**##**^	4.49 ± 0.34^∗∗^	3.66 ± 0.27^∗∗^	2.59 ± 0.21^∗∗^
MDA (nM/mg pro)	7.13 ± 1.38	19.34 ± 2.40^**##**^	15.38 ± 1.34^∗∗^	11.49 ± 1.18^∗∗^	8.52 ± 1.96^∗∗^
LDH (U/L)	643.82 ± 26.17	1205.50 ± 69.82^**##**^	940.74 ± 32.66^∗∗^	840.44 ± 31.39^∗∗^	758.46 ± 29.14^∗∗^
CK (U/mg pro)	0.55 ± 0.13	1.29 ± 0.16^**##**^	0.80 ± 0.11^∗∗^	0.70 ± 0.06^∗∗^	0.59 ± 0.08^∗∗^
TNF-*α* (pg/mL)	4.63 ± 0.28	69.50 ± 6.71^**##**^	43.79 ± 3.50^∗∗^	23.70 ± 2.07^∗∗^	15.22 ± 3.22^∗∗^
IL-1*β* (pg/mL)	123.22 ± 15.09	1157.76 ± 63.51^**##**^	766.80 ± 61.68^∗∗^	468.69 ± 53.03^∗∗^	374.28 ± 47.04^∗∗^
IL-6 (pg/mL)	25.07 ± 2.66	545.68 ± 33.40^**##**^	251.92 ± 29.88^∗∗^	155.40 ± 25.81^∗^	89.86 ± 21.33^∗∗^

Values were expressed as mean ± S.D. (*n* = 8). ^##^*P* < 0.01 vs. the control group; ^∗^*P* < 0.05, ^∗∗^*P* < 0.01 vs. the PQ group.

**Table 3:** Effects of GC on antioxidant enzymes and inflammatory cytokines in PQ-induced RLE-6TN model after Nrf2 gene silencing.

Group	Control	PQ	PQ + GC		
Dose (*μ*M)		20	1	10	100
GSH (mg/g pro)	2.62 ± 0.10	1.48 ± 0.37^**##**^	1.51 ± 0.27	1.63 ± 0.19	1.54 ± 0.25
NADPH (*μ*M)	11.75 ± 1.62	23.88 ± 2.08^**##**^	24.22 ± 3.49	26.86 ± 2.48	25.23 ± 3.06
SOD (U/mg pro)	359.35 ± 24.58	143.99 ± 22.54^**##**^	155.19 ± 32.02	150.65 ± 29.62	159.66 ± 23.47
CAT (U/mL)	6.08 ± 1.39	1.61 ± 0.25^**##**^	1.63 ± 0.34	1.69 ± 0.13	1.49 ± 0.22
MDA (nM/mg pro)	9.05 ± 2.78	25.93 ± 2.73^**##**^	25.30 ± 2.76	23.46 ± 1.90	23.78 ± 3.60
LDH (U/L)	556.27 ± 26.89	1129.16 ± 94.37^**##**^	1123.00 ± 100.85	1100.44 ± 96.97	1078.73 ± 77.58
CK (U/mg pro)	0.44 ± 0.08	1.33 ± 0.14^**##**^	1.15 ± 0.24	1.21 ± 0.12	1.22 ± 0.12
TNF-*α* (pg/mL)	4.56 ± 0.32	68.97 ± 5.28^**##**^	71.56 ± 6.23	71.36 ± 6.39	66.60 ± 4.00
IL-1*β* (pg/mL)	119.76 ± 15.94	1163.34 ± 102.87^**##**^	1177.40 ± 60.43	1182.55 ± 97.18	1150.53 ± 108.70
IL-6 (pg/mL)	23.22 ± 3.07	543.87 ± 31.50^**##**^	523.05 ± 44.95	538.99 ± 40.40	518.22 ± 52.04

Values were expressed as mean ± S.D. (*n* = 8). ^##^*P* < 0.01 vs. the control group.

## Data Availability

All the data could be provided if qualified authors required it.
